# Identification and Quantification of Transient Receptor Potential Vanilloid 1 (TRPV1) in Equine Articular Tissue

**DOI:** 10.3390/ani10030506

**Published:** 2020-03-18

**Authors:** Anne Frank Gallagher vom Braucke, Nanna Lysemose Frederiksen, Lise Charlotte Berg, Stacie Aarsvold, Felix Christoph Müller, Mikael Ploug Boesen, Casper Lindegaard

**Affiliations:** 1 Large Animal Teaching Hospital, Department of Veterinary Clinical Sciences, Faculty of Health and Medical Sciences, University of Copenhagen, DK-2630 Taastrup, Denmark; rpw642@alumni.ku.dk (A.F.G.v.B.); djf458@alumni.ku.dk (N.L.F.); lcb@sund.ku.dk (L.C.B.); 2 Puchalski Equine Imaging, CA, Petaluma, CA 94954, USA; saarsvolddvm@gmail.com; 3 Department of Radiology, Herlev and Gentofte Hospital, DK-2730 Herlev, Denmark; christoph.felix.mueller@regionh.dk; 4 Department of Radiology, Copenhagen University Hospital Bispebjerg and Frederiksberg, DK-2400 Copenhagen, Denmark; Mikael.ploug.boesen@regionh.dk

**Keywords:** TRPV1, osteoarthritis, capsaicin, WORMS

## Abstract

**Simple summary:**

Osteoarthritis is affecting several species including the horse. Transient receptor potential vanilloid 1 (TRPV1)—also known as the ‘chili receptor’—is currently being investigated as a target for treating osteoarthritis in humans. To evaluate whether it could be a potential target in treating osteoarthritis in horses, we collected synovial membrane samples from healthy horse joints and horse joints with joint disease to investigate the expression of the TRPV1. Laboratory analysis showed that TRPV1 is present in horse joints, and that the levels might be elevated in diseased joints, even at a chronic state of disease. This means that TRPV1 could be used as a potential target for osteoarthritis treatment in horses. Future studies in this area will not only be beneficial to horses and their owners, but more knowledge about TRPV1, the mode of action, and possible side effects will also benefit translation into human osteoarthritis research.

**Abstract:**

Joint pain and osteoarthritis (OA) are some of the most common causes of lameness in horses, and most of the available treatments focus on symptomatic relief without a disease-modifying effect. TRPV1 is a potential target for treating joint diseases, including OA, and the present study aims to investigate if the TRPV1 receptor is present in equine articular tissue and determine whether the number of receptors is upregulated in joint inflammation. Metacarpo/metatarsophalangeal (MCP/MTP) joints from 15 horses euthanised for reasons unrelated to this study were included. Based on synovial fluid analysis, macroscopic evaluation, and magnetic resonance imaging (MRI), joints were divided into two groups: healthy joints and joints with pathology. ELISA analysis was performed on synovial tissue harvested from all joints. TPRV1 was found in all joints. The mean concentration of TRPV1 compared to total protein in healthy joints (8.4 × 10^−7^ ng/mL) and joints with pathology (12.9 × 10^−7^ ng/mL) differed significantly (*p* = 0.01, *t*-test with Welch correction). Quantitative real-time reverse transcriptase PCR analysis was performed on RNA isolates from synovial tissue from all joints. TRPV1 mRNA expression ratio normalized to glyceraldehyde 3-phosphate dehydrogenase (GAPDH) in healthy joints (0.16 (SD: 0.19)) and joints with pathology (0.24 (SD: 0.14)) did not differ significantly (*p* = 0.43, *t*-test with Welch correction). mRNA expression of interleukin-6 (IL-6) and tumor necrosis factor alpha (TNF-α) was very low for both groups. In conclusion, TRPV1 was detected both on mRNA and the protein level, with a higher expression of TRPV1 in samples from joints with pathology. Future studies will determine the clinical potential of equine TRPV1 as a target in the management of joint pain and inflammation.

## 1. Introduction

Joint pain and osteoarthritis (OA) are some of the most commonly reported causes of lameness and retirement in horses. One study showed that in a population of 797 horses and ponies from Great Britain, 13.9% suffered from symptomatic OA [1]. Equine OA is a disease process in synovial joints characterised by the destruction of articular cartilage, subchondral bone sclerosis, and marginal osteophyte formation, often accompanied by joint effusion and synovitis [2,3]. The most commonly described aetiology is trauma—either as a single event or as a series of microtrauma. This will induce an inflammatory response, which, in turn, will drive the process of articular cartilage breakdown and remodelling of the surrounding bone [4]. Consequently, horses diagnosed with OA are often retired or euthanised.

There is a broad spectrum of treatments for equine OA. Most of them are anti-inflammatory and pain-relieving without a true disease-modifying effect, although regenerative medicine in regard to equine OA is a rapidly evolving area [5,6,7,8]. Two of the drug groups most commonly used for treating the symptoms related to equine OA are nonsteroidal anti-inflammatory drugs (NSAIDs) and intra-articular corticosteroids, often in combination. NSAIDs are predisposing for gastrointestinal ulceration and are potentially nephrotoxic, and corticosteroids are potentially chondrotoxic [9,10,11,12]. In human medicine, so-called disease-modifying osteoarthritis drugs (DMOADs) are being investigated in an attempt to stop the disease progression, as well as being pain-relieving and anti-inflammatory. However, an effective disease-modifying drug has not yet been developed [13].

Several authors point to the transient receptor potential vanilloid 1 (TRPV1) ion channel as a possible target for new ways of treating joint disease including OA [14,15,16]. TRPV1 is a nonselective cation channel which is expressed in various tissues. It is also known as the “chili receptor” and is primarily activated by capsaicin, which is the pungent substance in chili peppers. Besides capsaicin, it can be activated by several other chemicals as well as heat, pH, and voltage [17,18,19,20]. Regarding joints and the sensory pathways related to these, TRPV1 has mainly been identified in C-type neurons, which account for a slower, deeper pain sensation than A-delta fibres [21]. TRPV1 receptors have been found in chondrocytes, macrophages, osteoclasts, osteoblasts and synovial fibroblasts [22,23,24,25,26], and it is generally established that activation of the receptor has a proinflammatory effect and that inhibition will lead to decreased pain and inflammation. [15,16].

Upregulation of TRPV1 in the articular tissue in connection with inflammation has been shown in multiple species, including humans, mice and rats, and the receptor has been proven to play an important role not only in the development of oedema and hyperalgesia, but also in the destruction of cartilage and surrounding bone, as seen in OA joints [15,26,27,28,29]. These results highlight the potential of TRPV1 as a promising target for future DMOADs.

Surprisingly, not only deactivation of TRPV1, but also prolonged activation of the receptor will lead to analgesia due to the depletion of neuropeptides and subsequent desensitization of the C-fibres [30], hence both TRPV1-antagonists and agonists have shown promising results in pain management [14,16,26,31]. This has been further reviewed by Kelly [15] and Galindo et al. [16]. Due to the above-mentioned findings, TRPV1 antagonists have been investigated both for their analgesic and anti-inflammatory properties [31,32]. However, systemic administration of the majority of the tested TRPV1 antagonists have caused hyperthermia in dogs and humans [32,33]. Intra-articular treatment might require a lower dosage with a subsequent reduced risk of side-effects [31]. Therefore, intra-articular treatment should be investigated further. 

TRPV1 has been suggested as a target for equine drugs, and mRNA coding for the receptor has been found in various equine nerves, but until now, the expression of TRPV1 in equine articular tissues has not been investigated [34,35]. Consequently, the aim of the present study was to identify the TRPV1 receptor in equine synovial tissue, and to investigate whether there was an upregulation in connection with joint pathology, as seen in other species.

## 2. Materials and Methods

This project was approved by the Ethical and Administrative Committee at Department of Veterinary Clinical Sciences, Faculty of Health and Medical Sciences, University of Copenhagen (2019-021). All horses were included after informed consent from the owner.

### 2.1. Animals and Sample Collection

Fifteen client-owned horses euthanised at the Large Animal Teaching Hospital, University of Copenhagen, for reasons unrelated to this research project, were included. All horses had to be >1 year of age [36] and the following information was registered for each horse: age, gender, breed, use, reason for euthanasia, history of lameness, previous synovial fluid analysis if applicable, previous diagnostic imaging if applicable. The population consisted of six mares and nine geldings between three and 25 years of age; the breeds included Icelandic horses (5), Danish Warmblood (3), Standardbred (2), Jutland Draft Horses (2), Quarter Horses (1), Norwegian Fjord horses (1), and a mixed-breed pony (1).

In total, 11 metacarpophalangeal and four metatarsophalangeal (MCP/MTP) joints were collected. After euthanasia, the distal limbs were removed at the carpal or tarsal joint to obtain the MCP/MTP joints. The limbs were stored at 5 °C until magnetic resonance imaging (MRI) was performed (within 48 hours after euthanasia). Synovial fluid from the MCP/MTP joints was collected into an EDTA tube and stored at 5 °C until analysis (within 24 h). After diagnostic imaging, the joint space was opened aseptically, macroscopic evaluation of the cartilage was performed, and digital photos were obtained to document joint status. The synovial membrane was harvested by dissection with rat tooth forceps and a scalpel, transferred into cryotubes, snap frozen in liquid nitrogen, and stored at −80 °C.

Joints were divided into three predefined groups (Table 1) based upon history, clinical examination, standard synovial fluid analysis, high-field MRI, and macroscopic evaluation of the joint surfaces—see detailed descriptions of methods below.

### 2.2. Magnetic Resonance Imaging

MRI was performed in a 3T high-field scanner (Magnetom Verio: Siemens Healthineers, Erlangen Germany) using a dedicated 15-channel transmit/receive knee coil. The MCP/MTP joints were placed in the center of the coil. The protocol used is listed in Table 2.

**Figure 1:**
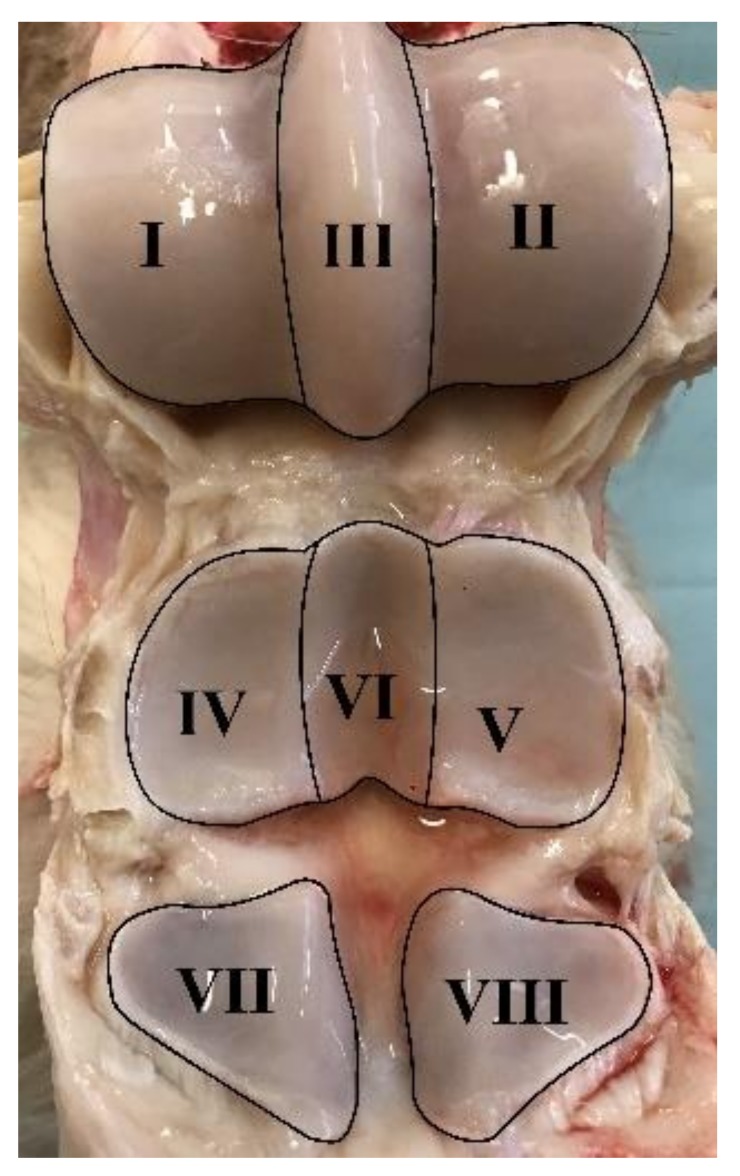
Regions defined in the metacarpophalangeal/ metatarsophalangeal joints.

All MR images were analysed by a board-certified specialist in veterinary diagnostic imaging (S.A., Diplomate of the American College of Veterinary Radiology) and scored using a whole organ MRI score (WORMS) modified from Smith et al. [40]. Eight regions were defined in the MCP/MTP joint: lateral and medial MC3/MT3 condyles (I–II), sagittal ridge (III), lateral and medial proximal phalangeal joint (P1) (IV–V), sagittal groove (VI), and lateral and medial sesamoids (VII–VIII) (Figure 1). Each region was scored using the criteria listed in Table 3 (0–19 points/region). Synovial thickening and joint effusion were given maximum of one point each for the entire joint. Final scores were summed to yield a WORMS for the entire MCP/MTP joint (0–154 points). A cut-off score was set at 10 points, i.e., WORMS >10 was regarded as OA.

### 2.3. Macroscopic Evaluation

Macroscopic evaluation was performed using a scoring system adapted from McIlwraith et al. [41]. Each of the eight regions described above in the MCP/MTP joint was scored using the system listed in Table 4. Each region was given between 0–6 points, and the scores were summed to yield an overall macroscopic evaluation score (0–48).

### 2.4. Synovial Fluid Analysis

Protein concentration (g/L) was measured with a refractometer (ATAGO Co. Ltd., Tokyo, Japan). Total Nucleated Cell Count (TNCC) was performed manually using a Bürker–Türk chamber (“Brigthline”, Marienfeld VWR, Søborg, Denmark). A line smear was stained using Hematoxylin and Eosin stain (HE) for future reference. A small portion of the synovial fluid was used for cytospin and subsequent smear, HE staining, and differential count. The rest of the synovial fluid was centrifuged for 10 min at 2500 *g*, and the supernatant was harvested and stored at −20 °C.

### 2.5. ELISA

Synovial tissue from all joints in the study was analysed using a commercially available ELISA assay (horse transient receptor potential cation channel subfamily V, member 1 (TRPV1) ELISA Kit, Cat.No.: MBS090478 from MyBioSource, San Diego, CA, USA) read with a Multiskan EX ELISA-reader (Thermo Fischer Scientific, Waltham, MA, USA). The synovial tissue was prepared according to the ELISA kit manual: 20 mg tissue was added to 200 µL PBS (Cat.No.: A9177.0100, Applichem GmbH, Darmstadt, Germany) and homogenised using an IKA T10 Basic Ultra-Turrax tissue homogeniser (IKA^®^-Werke GmbH & Co., Staufen, Germany) while kept below 10 °C. The homogenate was centrifuged for approximately 20 min at 1020× *g* at 4 °C and the supernatant was collected. Total protein concentration was determined by optical density measurement (NanoDrop TM Spectrophotometer (Thermo Fischer Scientific, Waltham, MA, USA)), and the samples were stored at −20 °C until analysis. Since TRPV1 is a cell membrane-associated receptor and in order to standardize for total amount of cells between samples, TRPV1 concentrations were normalized to total protein concentration for each sample, yielding results organised as the TRPV1 concentration as a ratio of the total protein concentration.

### 2.6. RNA Isolation and Quantitative Real-Time Reverse Transcriptase PCR Analysis

Synovial membrane tissue (60 mg) was lysed in 1 mL TRI Reagent (Molecular Research Centre, Inc. Cincinnati, OH, USA) and homogenised using an IKA T10 Basic Ultra-Turrax tissue homogeniser while kept below 10 °C. The homogenate was phase separated by adding 0.2 mL chloroform (Cat. No.: 24.751.000, Th. Geyer, Roskilde, Denmark), shaken vigorously for 15 seconds, allowed to stand for 15 min at room temperature (RT), and centrifuged at 12,000× *g* for 15 min at 4 °C. The upper phase containing the RNA was transferred to a fresh tube. RNA was precipitated by adding 0.5 mL 2-propanol (Cat.No.: 11.361.000, Th. Geyer, Roskilde, Denmark), incubated for 8 min at RT, followed by centrifugation at 12,000× *g* for 8 min at 4 °C. After removing the supernatant, the RNA pellet was washed by adding 1 mL 75% ethanol (Cat.No.: 698191, Glostrup Pharmacy, Glostrup, Denmark) and centrifugation at 7500× *g* for 5 min at 4 °C. The supernatant was removed, and the pellet was air dried for 5–7 min. The pellet was resuspended in 70 µL double-distilled water and incubated for 10 min at 60 °C. Total RNA concentration was determined by optical density measurement (NanoDrop TM Spectrophotometer), and total RNA isolates were kept at −80 °C until further processing.

cDNA was synthesized from 1.0 μg total RNA. Reverse transcriptase PCR mastermix (Promega, Madison, WI, USA) consisted of 5 μL RT buffer, 1.3 μL dNTP mix (10 μM) (Thermo Fischer Scientific, Waltham, MA, USA), 0.25 μL random hexamer primers (2 μg/μL) (TAG Copenhagen, Copenhagen, Denmark), 0.25 μL Oligo-dT primers (0.5 μg/μL) (TAG Copenhagen, Copenhagen, Denmark), 0.8 μL RNasin^®^ Plus RNase inhibitor (40 U/μL) (Promega, Madison, WI, USA), 1 μL M-MLV Reverse Transcriptase (200 U/μL) (Promega, Madison, WI, USA), and sterile water. Reverse transcription was performed in a BIOmetra^®^ T-Gradient thermocycler (Thermo Fischer Scientific, Waltham, MA, USA) at 25 °C for 10 min, 42 °C for 60 min, and 95 °C for 5 min. Samples were stored at −20 °C.

Species-specific intron-spanning equine primers were used to amplify TRPV1, interleukin-6 (IL-6), tumor necrosis factor alpha (TNF-α) and glyceraldehyde 3-phosphate dehydrogenase (GAPDH) (reference gene). Primers are listed in Table 5. Quantitative real-time reverse transcriptase PCR (qPCR) was performed in triplicates using LightCycler^®^ Fast Start DNA Master SYBR Green I and LightCycler^®^ Real-Time PCR System (Roche, Basel, Switzerland). Results are presented as relative quantitative expression ratios between the target genes (TRPV1, IL-6, TNF-α) and reference gene (GAPDH).

### 2.7. Statistics

Data sets were tested for normality using a Shapiro–Wilks test, and subsequently an unpaired t-test with assumed Gaussian distribution was used to analyse for differences between groups. Welch correction was applied. All data analysis was performed using GraphPad Prism version 8.00 for Windows [44]. The significance level was set at *p* < 0.05.

## 3. Results

### 3.1. Division into Groups

**Figure 2:**
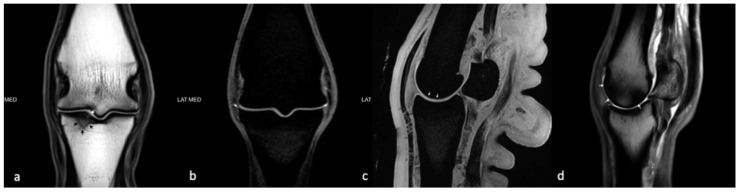
Image panel representative of some of the MRI findings from the study. (**a**) Dorsal plane T1W TSE image at the mid aspect of the metacarpophalangeal joint of horse #7 showing a fissure within the subchondral bone plate of the proximal phalanx along the medial aspect of the sagittal groove (**white arrows**) that is surrounded by sclerosis and intra-osseous fluid accumulation (black arrows). (**b**) Dorsal plane T1W VIBE image of horse #9 near the dorsal aspect of the metacarpophalangeal joint showing osteophytes arising from the dorsal, proximal, abaxial margins of the proximal phalanx (**white arrows**). (**c**) Sagittal plane T1W VIBE image at the mid aspect of the medial third metacarpal condyle showing irregularity of the subchondral bone plate of the third metacarpus and signal alteration of the overlying articular cartilage (**white arrows**). (**d**) Sagittal plane PDW TSE image of the medial aspect of the third metacarpal condyle showing regions of low signal bone intensity of the dorsal and palmar aspects of the condyle as indicated by the white arrows.

**Figure 3:**
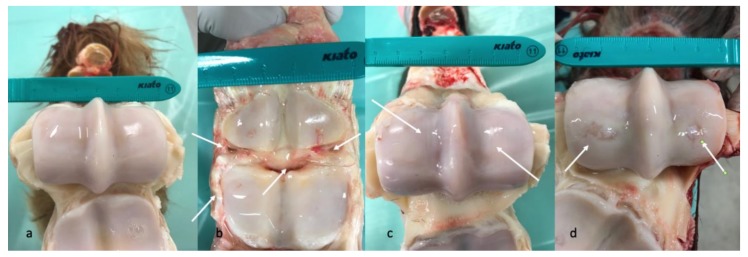
Image panel representative of some of the findings from the macroscopic pathology evaluations described in the study. (**a**) The opened fetlock of horse #6 showing smooth glistening cartilage surfaces with no synovial hyperplasia and no signs of wear lines or erosions. (**b**) The opened fetlock of horse #3 showing a moderate to marked degree of synovial hyperplasia (**white arrows**). (**c**) The opened fetlock of horse #14 showing a mild to moderate degree of superficial wear lines on both condyles of the third metacarpal bone (**white arrows**). (**d**) The opened fetlock of horse #4 showing severe full thickness erosions of the cartilage of the central distal parts of the medial and lateral condyles of the third metacarpal bone (**white arrows**).

Using the criteria listed in Table 1, the horses were divided into following groups:

A detailed list of results for each horse can be seen in Appendix A. Due to only one horse being in group C, groups B and C were pooled (all joints with pathology) and compared to group A (all healthy joints) for all analyses (Table 6). Age differed significantly between Group A (mean age 8.3 years) and Group B (mean age 15.3 years) (*p* = 0.03), and between group A and groups B and C (mean age 15.4 years) (*p* = 0.02). Representative images of MRI and macroscopic findings are shown in Figure 2 and Figure 3.

### 3.2. Levels of TRPV1 in Metacarpo-Phalangeal/Metatarso-Phalangeal (MCP/MTP) Joints

**Figure 4:**
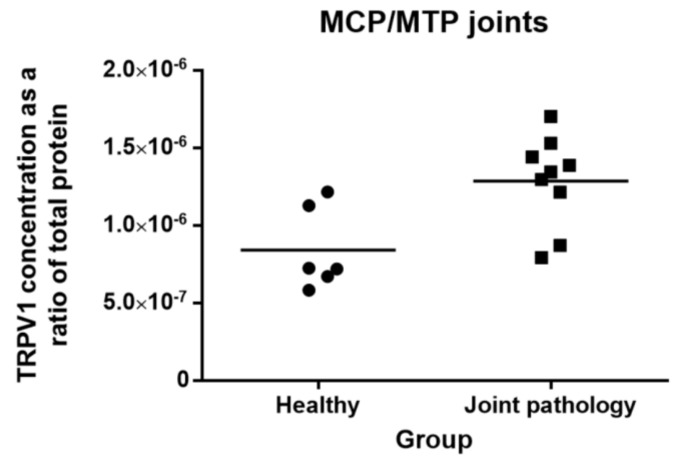
Concentration of transient receptor potential vanilloid 1 (TRPV1) protein in synovial membrane tissue from MCP/MTP joints (ng/mL) relative to total protein (ng/mL). Symbols indicate individual joints (circles = healthy; squares = joint pathology). The mean is indicated by the horizontal bar.

TRPV1 receptor was detected in all equine synovial tissue samples. TRPV1 concentration as a ratio of total protein in joints with pathology (mean: 12.9 × 10^−7^ (SD: 2.9)) differed significantly from healthy joints (mean: 8.66 × 10^−7^ (SD: 3.02)) (*p* = 0.01) (Figure 4). Comparing group A and B horses without including the one group C horse does not change the results (*p* = 0.03).

### 3.3. Levels of mRNA Coding for TRPV1, IL-6 and TNF-α in MCP/MTP Joints

**Figure 5:**
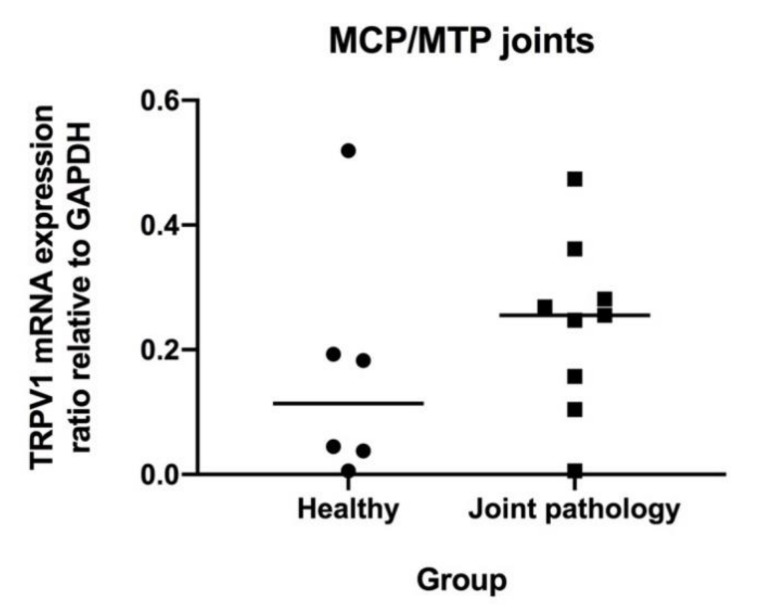
TRPV1 mRNA expression ratio relative to glyceraldehyde 3-phosphate dehydrogenase (GAPDH) in synovial membrane tissue from metacarpo-phalangeal/metatarso-phalangeal (MCP/MTP) joints. Symbols indicate individual joints (circles = healthy; squares = joint pathology). Mean is indicated by horizontal bar.

TRPV1 mRNA was detected in all equine synovial tissue samples. TRPV1 expression ratios normalized to GAPDH in joints with pathology (mean: 0.24 (SD: 0.14)) did not differ significantly from healthy joints (mean: 0.16 (SD: 0.19)) (*p* = 0.43) (Figure 5). Comparing group A and B horses without including the one group C horse does not change this result (*p* = 0.45). mRNA expression of IL-6 and TNF-α was very low for both groups.

## 4. Discussion

The present study demonstrated that TRPV1 was present as mRNA and protein in the equine synovial membrane in healthy and diseased joints. These results are consistent with previous studies, where TRPV1 has been found in synovial tissue from several other species [26,27,28], and in both neural and respiratory tissue in the horse [45].

Regulation of TRPV1 in diarthrodial joints has been studied in rats and humans. In a mono-iodo-acetate-induced (MIA) arthritis model in rats, 71% of sensory neurons stained positive for TRPV1 when using immunohistochemistry, compared to 54.3% of sensory neurons in control rats injected with saline [27]. Another study compared TRPV1 immunoreactivity in synovial sections from human patients with OA, rheumatoid arthritis (RA), and post mortem (PM) controls without known joint pathology, and showed an increased fraction of TRPV1 immunoreactivity in synovial sections from patients with OA (7.3%) and RA (5.2%) compared to PM controls (0.0%) [26]. The present study revealed a significant upregulation of TRPV1 protein concentration in horses with joint pathology compared to healthy controls. However, as a similar difference could not be shown for TRPV1 mRNA, the conclusion regarding upregulation during disease is not unequivocal, although visual inspection of Figure 5 seems to indicate a tendency towards a higher level in the group with joint pathology.

One factor that may have affected the results in the present study is the very heterogenous nature of the diseased group. Horses were euthanised for other reasons than this research project, and were therefore not selected for symptomatic and/or longstanding joint problems. Consequently, only very few included horses had a clinical history of diagnosed joint pathology, and assignment into the pathology group was based solely on macroscopic and MRI findings. This differs from other studies regarding TRPV-1 expression [27]. None of the joints in the present study scored maximum points in the WORMS or during the macroscopic evaluation and are therefore considered less affected by intra-articular inflammation than the MIA-induced joints in the study by Fernihough et al. [27]. The methods and criteria used for categorization of joint disease status are criteria used both in the literature and in the clinic. We therefore believe this to be a clinically relevant group of horses, representing the stage just before the need for intervention. However, further studies including horses with clinical disease and lameness might elucidate whether TRPV1 is truly upregulated during disease.

The synovial membrane is a highly reactive tissue that changes not only in cellular activity, but also in morphology when reacting to inflammation. The reactive synovial lining will increase from only two to three cell layers in the healthy joint to multiple layers in the inflamed joint. This is a challenge for any study working with harvested synovial tissue especially from healthy joints due to the difficulty in separating the synovial layer from the sub-synovial tissue, as acknowledged by other studies [40]. In this study, the synovial tissue was harvested carefully using rat tooth forceps and a scalpel, relying on the gross appearance of the synovial tissue, which has a “fatty”, shiny presentation. Another method described is to use tissue shavers to collect synovial tissue, but this has mostly been done in joints with synovial hyperplasia and, hence, more cell layers [46]. Since the cell density in the synovial lining is very dense, with an almost epithelial appearance compared to the more fibrous and far less cell dense sub-synovial tissue, the risk of this affecting the overall results is considered low.

In the present study, MRI was used to assess joint pathology before dividing joints into groups. MRI has been found to be superior to radiography, when assessing non-cartilaginous changes in equine MCP joints with OA using macroscopic and histological evaluations as the gold standard [47,48]. Furthermore, MRI has been found to be considerably more sensitive than radiography when evaluating osteophytes, fissure fractures, subchondral bone damage, trabecular bone damage, or when discriminating joint effusion from soft tissue thickening [49,50]. Other studies have investigated the use of MRI in horses with lameness located to the MCP/MTP region without radiographic evidence of pathology. Most of these horses were successfully diagnosed using MRI, including horses with OA, chronic subchondral bone injury, fracture pathology or osteochondral defects, yet without a sufficient change in bone density to be observed on radiographs [51,52,53,54].

Most studies using MRI in horses have been performed in low- or high-field scanners ranging from 0.25–1.5 Tesla. The scanner used in the present study was a 3 Tesla high-field scanner with protocols adjusted to this magnetic flux density, making it capable of producing more detailed images (Figure 2). The OA changes on the MR images were scored using a modified whole organ MRI score (WORMS) based on an article by Smith et al. [40], who developed a similar score for the equine carpal joint. The WORMS used in the present study only included characteristics deemed statistically significant by Smith et al. [40], but the use of WORMS in equine imaging is still new, and the method is not yet validated. To the authors’ knowledge, no studies have determined a WORMS cut-off value for equine OA. In human medicine, WORMS is used as a research tool to grade osteoarthritic changes, but a cut-off value has yet to be defined [55]. Since it was not possible to extrapolate a cut-off value to equine MCP/MTP joints, the final cut-off value was determined based on a comparison with the macroscopic evaluation. All joints were macroscopically assessed when opened. Synovial hyperplasia, fibrillation, wear lines, and cartilage defects were noted, and photographic images recorded following a standardized protocol were obtained and saved for reference (Figure 3). In general, there was good conformity between the MRI evaluation and the macroscopic evaluation (Appendix A).

The therapeutic role of TRPV1 is a quickly emerging subject in both human and veterinary medicine, and some authors have even referred to it as “the holy grail of pain management” [56]. Time and future studies will tell whether TRPV1 agonists or antagonists, alone or in combination with other drugs, will be an efficient way to treat pain and inflammation in joints as well as stopping the deleterious effects of equine OA. For future studies, we recommend a larger population of horses representing two groups: horses with no musculoskeletal problems and no OA changes, and horses which have been diagnosed with clinically manifested, symptomatic OA before euthanasia. This would ensure a more well-defined group of horses with symptomatic joint pathology. We also recommend further investigation of the presence and regulation of TRPV1 in the articular tissues, including the cartilage. Furthermore, if the future goal is to use the TRPV1 as a target for DMOADs, the next step will be to test the clinical response to either agonists or antagonists in horses. One study in dogs has shown very promising results in regard to efficient pain management with few or no side effects, when treating naturally occurring OA with a single intraarticular injection of resiniferatoxin, a TRPV1 agonist [31]. This protocol could easily be adapted for equine use and clinical trials could be undertaken.

In conclusion, the results from the present study show that TRPV1 is present in equine synovial membrane. It was not possible to unequivocally determine whether TRPV1 expression is upregulated in joints with pathology. Our results create a sound foundation for further investigations into TRPV1 as a local target for treatment of OA in horses.

## Figures and Tables

**Table 1:** Categorization of joint disease and division of horses into three groups (total nucleated cell count (TNCC); magnetic resonance imaging (MRI); whole organ MRI score (WORMS); Ymetacarpophalangeal (MCP); metatarsophalangeal (MTP)).

No Joint Pathology (Group A)	Osteoarthritis (Group B)	Arthritis (Group C)
- Minimum 1 year of age [36] and - No known history of lameness of the limb in question and - Normal synovial fluid analysis (clear yellow, viscous, protein < 20 g/L, TNCC < 1 × 10^9^/L, <10% neutrophils) (adapted from [37]) and - No changes on MRI compatible with chronic arthrosis, i.e., WORMS <10 points and - Osteoarthritic changes graded <4 points during macroscopic evaluation of the joint	- A history of continuous or non-continuous lameness >3 months of the limb in question and/or - Positive MCP/MTP joint block or nerve blocks affecting this joint during the diagnostic work-up and/or - Synovial fluid can have increased TNCC but it should mainly be mononuclear cells, i.e., not compatible with acute arthritis (92) and/or - Changes on MRI compatible with osteoarthritis, i.e., WORMS graded 10 points or above and/or - Osteoarthritic changes graded four points or above during macroscopic evaluation of the joint	- Symptoms (acute lameness, effusion) of 1–7 days’ duration and - Synovial fluid compatible with acute synovitis (TNCC > 1 × 10^9^/L and/or protein >25 g/L) and/or >85% neutrophils in cases with septic arthritis [38,39] and - No changes on MRI compatible with Osteoarthritis, i.e., WORMS <10 points and - Osteoarthritic changes graded <4 points during macroscopic evaluation of the joint

**Table 2:** Magnetic resonance sequences used. Repetition time (TR); echo time (TE); inversion time (TI); flip angle (FA); field of view (FOV); pixel (Px); number of excitations (NEX).

	TR (ms)	TE (ms)	TI (ms)	Slice Thickness (mm)	FA (°)	FOV (mm)	Echo Train length	Bandwidth (Hz/Px)	Matrix	NEX	Scan Time (min)
**Axial_fse_blade** **_fatsat**	5000	26	-	2.5	145	160	9	318	384 × 384	2	06:02
**Coronal_fse_T1W**	700	16	-	2.5	150	160	3	260	224 × 320	2	02:09
**Coronal_fse_T2W** **_fatsat**	5000	61	-	2.5	150	160	11	220	224 × 320	2	06:42
**Coronal_STIR**	4900	32	220	2.5	150	160	10	252	224 × 320	2	02:53
**Sagittal_fse_PDW**	5000	19	-	2.5	150	160	8	407	307 × 384	1	06:32
**Sagittal_fse_T2W**	5300	55	-	2.5	150	160	8	328	307 × 384	1	06:34
**T1W_Vibe_ISO** **_3D**	11.6	05:39	-	0.6	10	160	-	190	256 × 256	1	03:45
**Total Scan Time:**											33:06

**Table 3:** Modified whole organ magnetic resonance score (WORMS) for the metacarpophalangeal/metatarsophalangeal joints.

Parameter Assessed	0	1	2	3	4
**High-signal bone lesions (PD-FS and T2-STIR), size**	None	<25% of region	25–50% of region	>50% of region	-
**High-signal bone lesions (PD-FS and T2-STIR), intensity**	None	Mild	Moderate	Marked	-
**Low-signal bone lesions (PD- and T2-weighted images)**	<10% of region	10–25% of region	26–50% of region	>50% of region	-
**Osteophyte formation, size of largest osteophyte in the region**	None	<1 mm	1–2 mm	>2 mm	-
**Cartilage signal abnormality (SPGR-FS and PD-FS)**	No abnormalities	Single lesion <5 mm	Single lesion 5–10 mm or multiple lesions < 5 mm	Single lesion > 10 mm or multiple lesions 5–10 mm	-
**Subchondral bone irregularity**	Smooth and regular chondro-osseus junction	Mild subchondral plate irregularity	Marked subchondral plate irregularity with intact trabecular bone	Bone irregularity extending to the trabecular bone with preservation of some trabecular pattern	Cyst-like formation

**Table 4:** Scoring system for macroscopic evaluation of equine joint surfaces [41].

Type of Pathology Assessed	0	1	2	3
**Wear lines**	None	1 or 2 partial-thickness wear lines	3–5 partial thickness or 1–2 full-thickness wearlines	>5 partial thickness or >2 full-thickness wear lines
**Erosions**	None	Partial-thickness erosion, <5 mm in diameter	Partial thickness erosion, >5 mm in diameter	Full thickness erosion

**Table 5:** Species-specific primers.

Primer Name	Forward Primer 5’–3’	Reverse Primer 5’–3’	Reference/Accession Number
TRPV1	TTG AGG ACG GGA AGA ATG AC	GAT GAG CAT GTT GAT CAG GA	Berg, LC/XM_014727972.2
IL-6	ATG GCA GAA AAA GAC GGA TG	GGG TCA GGG GTG GTT ACT TC	Haneda et al. [42]
TNFα	GAG GGA AGA GCA GTT ACC GAA TG	GGC TAC AGG CTT GTC ACT TGG	Berg, LC/NM_001081819.2
GAPDH	GGG TGG AGC CAA AAG GGT CAT CAT	AGC TTT CTC CAG GCG GCA GGT CAG	Iqbal et al. [43]

**Table 6:** Number of horses in groups A, B, and C.

	No Joint Pathology (A)	Chronic Arthrosis (B)	Acute Arthritis (C)
**Number of Horses**	6	8	1

## Appendix A

**Table A1:** Combined results from synovial fluid analysis, WORMS score and macroscopic evaluation of the metacarpo/metatarsophalangeal joints.

	Joint Evaluation		Synovial Fluid Evaluation			ELISA	qPCR	
Horse no.	Macroscopic Evaluation (0–56)	WORMS Total (0-154) and Max Regional Score (0–19)	Protein (g/L)	TNCC (×10^9^/L)	Differential Count (% Neutrophils of TNCC)	TRPV1 Concentration (ng/mL) Relative to Total Protein (ng/mL)	TRPV1 mRNA Expression Relative to GAPDH	Group
1	0	6/2 *	11	0.8	1.5	5.82 × 10^−7^	0.183	A
2	1	8/2 *	10	0.3	2.3	7.25 × 10^−7^	0.038	A
3	4	3/1 *	4	1.6	1.2	7.92 × 10^−7^	0.269	B
4	8	17/5	5	0.3	1.0	13.5 × 10^−7^	0.474	B
5	3	3/2	14	3.8	2.0	14.4 × 10^−7^	0.247	C
6	0	6/1	9	0.9	0.0	7.20 × 10^−7^	0.045	A
7	1	20/10	17	0.3	1.0	13.9 × 10^−7^	0.157	B
8	6	11/4	2	0.7	2.0	15.3 × 10^−7^	0.281	B
9	2	11/3	14	0.9	0.0	8.71 × 10^−7^	0.104	B
10	4	6/2	22	4.7	0.5	17.0 × 10^−7^	0.362	B
11	5	11/4	17	1.3	1.0	12.1 × 10^−7^	0.006	B
12	1	6/1	1	0.4	0.0	6.71 × 10^−7^	0.006	A
13	2	6/2	9	0.4	5.0 **	12.2 × 10^−7^	0.193	A
14	8	8/2	5	4.1	3.0	13.0 × 10^−7^	0.255	B
15	0	3/1	8	0.7	2.0	12.8 × 10^−7^	0.520	A

* Gas was present in these joints, hence the evaluation of cartilage signal abnormality on MRI was impaired. Cartilaginous changes seen during the macroscopic evaluation were given emphasis when determining which groups these horses were assigned to. ** Very few cells were present in this sample, hence the validity of this differential count is uncertain.
